# Prospective Memory Impairment and Executive Dysfunction in Prefrontal Lobe Damaged Patients: Is There a Causal Relationship?

**DOI:** 10.1155/2014/168496

**Published:** 2014-03-09

**Authors:** Giovanni A. Carlesimo, Margherita di Paola, Lucia Fadda, Carlo Caltagirone, Alberto Costa

**Affiliations:** ^1^Clinica Neurologica, Università Tor Vergata, Via Orazio Raimondo 18, 00173 Rome, Italy; ^2^Department of Clinical and Behavioural Neurology, IRCCS Santa Lucia Foundation, Via Ardeatina 306, 00179 Rome, Italy; ^3^Dipartimento di Medicina Clinica, Sanitá Pubblica, Scienze della Vita e dell'Ambiente, Piazzale Salvatore Tommasi 1, L'Aquila, Italy

## Abstract

*Background*. The prospective memory (PM) construct is aimed at capturing cognitive operations involved in the successful accomplishment of delayed intentions. It is generally agreed that PM impairment occurs in patients with prefrontal lobes damage. *Objective*. To evaluate if there is a causal role of a deficit of executive abilities (failures of planning, set-shifting, selective attention, or working memory) over the PM impairment. *Methods*. We report a detailed investigation of PM and executive abilities in two patients with posttraumatic damage to prefrontal lobes who complained from a reduced compliance with appointments and daily routines. *Results*. Laboratory tests confirmed a difficulty in fulfilling delayed intentions in response to the occurrence of critical events and elapsed time. In one patient, PM impairment was associated with poor performance on tests investigating planning, working memory, and mental shifting. The other patient performed in the normal range on all executive tests. *Conclusions*. Despite the frequent claim of a dependence of PM deficits from executive dysfunction, the reported cases demonstrate that this is not necessarily the case. The results are discussed in the light of current hypotheses relating PM impairment to other deficits that commonly occur as a result of damage to the prefrontal lobes.

## 1. Introduction

While engaged in the activities of daily living, people frequently need to keep in mind the intention to perform some actions in the future or in response to some event (e.g., buy some items at the grocery store on the way home from work) or at a certain time (e.g., attend a meeting at 11 o'clock). The ability to correctly fulfill delayed intentions is essential for independent human behaviour [1] and its deficit in brain-damaged individuals is severely disabling and responsible for many of their difficulties and concerns (e.g., missing appointments or not taking medication at the correct time) [2].

The theoretical construct of prospective memory (PM) is aimed at capturing the variety of cognitive operations involved in the successful accomplishment of delayed intentions. According to several authors [3, 4], a PM task is typically articulated into three main successive phases. In the first phase, a plan of future actions is formulated. The complexity of the plan varies according to many factors, including the number of actions to be performed and the chronological order imposed by the functional relationship between individual actions and some temporal or spatial constraints. After the plan has been formulated, a delay is usually interposed before the critical time or event for carrying out the intention(s) actually arrives. During this period, the plan has to be kept in memory and actively recalled. The individual should also be involved in some type of time monitoring and/or external world check so that he will not miss the critical time or event triggering realization of the intention(s). The last phase of a PM task starts when the individual recognizes that the critical time has elapsed or that the cue event has occurred. Thus, he is required to transitorily interrupt ongoing activity to perform the intended action(s). Following action completion and/or according to some intervening unforeseen occurrence, the prospective plan may need to be updated or possibly reformulated in terms of priorities and/or sequencing.

Correct fulfillment of the cognitive operations listed above requires the normal functioning and reciprocal integration of a variety of cognitive functions mediated by discrete cortical regions in the human brain. Many of these functions, such as planning, working memory (required to keep active and/or rehearse the intention over short periods), selective and divided attention (to manage the ongoing task while rehearsing the prospective intention and monitoring the passing of time or occurrence of the cue event), and mental shifting and task initiation (to promptly abandon the concurrent activity and initiate the PM task at the occurrence of the target event or when the time expires) are typically included in the executive functions domain [3, 5–7]. It is generally agreed that the neural circuits underlying executive functioning greatly involve the frontal lobes (e.g., [8]). In particular, both neuropsychological and functional neuroimaging data indicate that the dorsolateral prefrontal cortex is particularly involved in planning [9], working memory [10], divided attention [11], and shifting aptitude [12] abilities, and that the dorsomesial prefrontal regions with contiguous anterior cingulate are critical for selective attention and resistance to interference [13]. Declarative memory is another cognitive function likely implicated in normal PM functioning [6, 14]. There is general agreement that declarative memory supports the so-called* retrospective* component of a PM task, which consists of encoding, retention, and retrieval of the plan, including the context to act (time or event) and the specific actions to be performed, which should be kept distinct from the* prospective* component (i.e., plan formation, context or time monitoring, task interruption and shifting, and updating). The brain regions that are critical for declarative memory are mainly located in the mesial temporal lobes and connected subcortical structures in the diencephalon [15].

The assumption that a PM impairment results from dysfunction of the prospective and/or retrospective component predicts that a patient with poor performance on PM tasks will also be impaired on neuropsychological tests assessing executive abilities or declarative memory. Indeed, a number of studies have documented this kind of relationship. In various etiological groups of brain-damaged individuals, a significant association was found between poor performance on PM tests and impaired planning [16], set-shifting abilities [17], selective and divided attention [18], and working memory. Shallice and Burgess [19] reported three cases of patients with large bilateral prefrontal damage who failed real life and laboratory tests that required the coordination and effective accomplishment of multiple tasks but performed substantially within the norm on several tests of executive functions (see, for similar a case, [20]). The authors concluded that planning failure was the main deficit in these patients, because it interfered with the ability to keep in mind and/or effectively accomplish the plan to perform multiple actions in the correct sequence (see also [21]).

Nevertheless, not all studies have found a straightforward relationship between the failure of executive abilities or declarative memory and PM impairment. In a recent meta-analytic review of PM functioning in patients with Alzheimer's disease or Mild Cognitive Impairment, PM was only modestly correlated with measures of retrospective memory (median *r* = 0.27) and executive functioning (median *r* = 0.30) [22]. In another study in Ecstasy users, long-delay prospective memory was not associated with retrospective memory or other aspects of executive functions [23]. Finally, in people with schizophrenia, event-based PM was associated with general intelligence but not measures of executive functions or declarative memory [24].

It is widely agreed that the brain site whose damage is responsible for PM impairment is in the anterior portions of the frontal lobes. Indeed, the above-reported single cases of selective impairment in multitasking conditions suffered from prefrontal focal damage [19, 20]. An association between focal damage to prefrontal lobes and PM impairment has also been reported in some group studies [25–27]. Furthermore, consistent data can also be found in the functional neuroimaging literature. Both PET and fMRI studies report a specific involvement of the polar areas of the frontal lobes corresponding with BA10 and possibly the relative specialization of more mesial regions in monitoring the external environment in order to detect target events and of lateral regions to maintain and retrieve the PM intention [28, 29].

The aim of the present study was to further investigate the role of executive functions in the genesis of PM impairment resulting from prefrontal damage. For this purpose, we report the cases of two patients with focal damage to anterior portions of the frontal lobes resulting from traumatic brain injury. Both patients had clear difficulty in fulfilling delayed intentions in response to the occurrence of critical events and elapsed time in real life and laboratory tests. In one patient, the PM impairment was associated with poor performance on a variety of tests investigating executive abilities, such as planning, working memory, and mental shifting. The other patient performed in the normal range on all tests of the executive battery. These results are discussed in the light of current hypotheses relating the impairment of PM abilities to other deficits that commonly occur as a result of damage to the prefrontal lobes.

## 2. Case Reports

### 2.1. Clinical History

V.B. is a right-handed twenty-four-year-old male. When he was seven years old, about 16 years before the present evaluation was performed, he suffered from a severe closed-head injury due to an in-home accident. In the aftermath, no significant behavioral or cognitive disorders were reported. In later time, the patient attended regular course of study and university achieving a bachelor degree in philosophy. The patient came to our ambulatory in March 2010 by reason of the onset of epileptic seizures. The patient referred that the main memory deficits were represented by the difficulty to attend to appointments and by topographic disorientation. Family members also report that V.B. presents with dysphoric mood and emergent awareness about his cognitive difficulties. Moreover, parents reported puerile behaviour that together with the attitude to jump to premature conclusions and grandiosity significantly affected social relationships.

At the time of our assessment V.B. was a fully alert, cooperative individual, without significant attentional difficulty. His spontaneous speech was fluent, syntactically and phonologically correct. A clinical examination and the administration of the Mini International Neuropsychiatric Inventory [30] did not evidence major psychopathological disorders. We performed an extensive neuropsychological assessment (see below for a detailed description of the neuropsychological tests battery administered) documenting the presence of prospective memory disorders, in the context of normal scores on all tests investigating the other cognitive domains. After the neuropsychological examination, V.B. entered in a cognitive rehabilitation program for PM disorders therapy. However, the prospective memory difficulties of the patient significantly affected his ability to correctly attend to the rehabilitation sessions. Indeed, these difficulties, mainly related to a failure to be in schedule with therapeutic treatment, led to the interruption of the treatment itself. In order to overcome above difficulties, the patient uses external aids (e.g., agenda and tablet) that supported him to follow his academic course. V.B. is able to discuss intelligently matters such as the philosophy or moral dilemmas but he was unable to inhibit inappropriate responses and to sustain behaviour without perseveration.

GP is a right-handed eighteen-year-old male who came at our observation in the February 2010, about one year after the occurrence of a pile-up wreaked severe closed head injury followed by a 20-day lasting coma. In the aftermath, GP returned to school and he is currently attending the last year of secondary school (accounts school). A cerebral CT scan performed in acute phase documented multiple contusions at the level of frontal and occipital lobes, posttraumatic subdural frontal-parietal haematoma, and spread edema. A later brain MRI showed an atrophic malacic area within frontal lobe, smaller similar areas at the level of right parasagittal and temporal-occipital lobes, reduced thickness of corpus callosum, and ventricles enlargement.

The patient was admitted to our hospital to attend a cognitive rehabilitation program. Indeed, GP complains of some difficulties to remember realizing future intentions and, particularly, to attend to appointments and to keep tasks in mind, in the context of no other significant cognitive deficits. These difficulties are confirmed by his parents who also report loss of spontaneity, curiosity, and initiative, with apathetic blunting of feeling. These aspects seem to particularly affect social relationships.

At the time of assessment GB did not suffer from posttraumatic amnesia any longer; he was fully alert and cooperative, without any apparent attentional difficulty. The Level of Cognitive Functioning Scale score was 8 indicating purposeful and appropriate responses. The patient underwent an extensive clinical neuropsychological examination (see below for a description of the tests administered) documenting reduced capacity of the declarative memory system and impaired ability to access own lexical-semantic knowledge. Clinical examination and the Mini International Neuropsychiatric Inventory administration did not evidence major psychopathological disorders. However, the patient shows foresight decrease. Indeed, he seemed scarcely interested in the outcome of the rehabilitative intervention as well as in the implications for his own life projects. At the time of assessment, GP does not present any other behavioural or affective disorder.

### 2.2. Neuroimaging

Both patients underwent MRI examination for the localization of brain damage. All MRI data were acquired on a 3T Allegra MRI system (Siemens, Erlangen, Germany) using a birdcage head coil. Scans were collected in a single session, with the following pulse sequences: (a) proton density (PD) and T2-weighted double turbo spin echo (SE) acquired in transverse planes (TR: 4500 ms, TE: 12 ms, TE: 112 ms, FOV 230 × 172 mm, matrix 320 × 240, slice thickness: 5 mm, number of slices: 24); (b) fluid-attenuated inversion recovery (FLAIR) in the same planes as the SE sequence (TR/TE/TI: 8500/109/2000 ms; FOV: 230 × 168 mm, matrix: 256 × 256, slice thickness: 5 mm, number of slices: 24); (c) T1-weighted 3D images, with partitions acquired in the sagittal plane, using a modified driven equilibrium Fourier transform (MDEFT) sequence (TE/TR/TI: 2.4/7.92/910 ms, flip angle: 15 degrees, 1 mm^3^ isotropic voxels).

**Figure 1:**
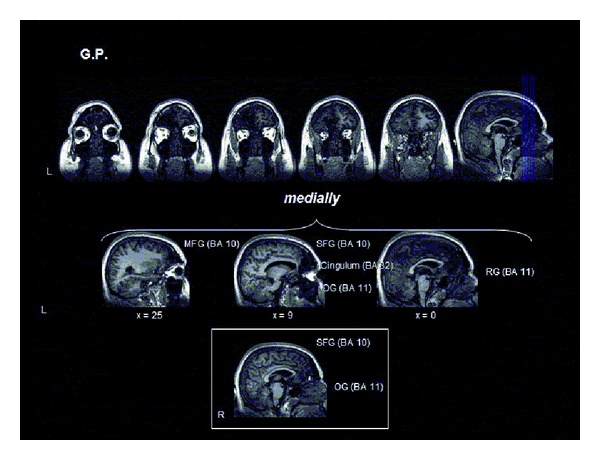
Localization of brain damage in G.P. See the text for details.

G.P. showed cortical-subcortical damage in the most anterior regions of the left and right frontal lobes. On the left, the damaged area involved both the polar and medial portion of the frontopolar cortex (BA 10) and the anterior cingulum (BA 32). Ventrally, damage included the left rectal and orbital gyri (BA 11). In the right hemisphere, tissue damage medially involved the frontopolar cortex (BA 10) and orbital gyrus (BA 11) (Figure 1).

**Figure 2:**
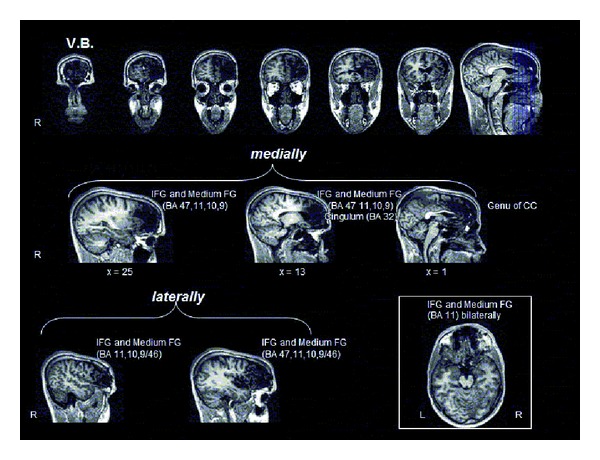
Localization of brain damage in V.B. See the text for details.

V.B. presented an area of cortical-subcortical damage at the level of the right frontal lobe. Medially, the damage involved the inferior frontal gyrus (BA 47) and the medium frontal gyrus (BA 11, 10, 9), the genu, and the white matter surrounding the corpus callosum (anterior cingulum BA 32). Laterally, proceeding rostrocaudally, damage involved the frontopolar (BA 10), dorsolateral (BA 9, 46, 9/46), and ventrolateral prefrontal cortex (BA 47) (Figure 2).

### 2.3. Neuropsychological Evaluation

V.B. and G.P. were submitted to a neuropsychological battery that included tests for the assessment of general intelligence on visual data (Raven's Coloured Progressive Matrices, [31]), visuospatial abilities (Copy of simple drawings freehand and with landmarks and Copy of Rey's Figure, [31, 32]), short-term memory and working memory for verbal and visuospatial data (Digit span and Corsi block span, [33]), episodic memory for verbal information (15-word immediate and delayed recall and Prose immediate and delayed recall, [32, 32]), and episodic memory for visuospatial data (immediate and delayed reproduction of Rey's Figure and Corsi supraspan sequence learning, [32, 34]).

The two patients were also administered a test battery to evaluate a variety of executive functions including set-shifting aptitudes (Modified Card Sorting Test, Trail-Making test, and alternate Word Fluency, [35–37]), phonological and semantic word fluency [31, 34], selective attention (Stroop test, [38]), planning abilities (The Zoo Map test, [39]), and working memory (Digit and Corsi span backwards, [40].

### 2.4. Prospective Memory Assessment

#### 2.4.1. Event-Based PM

The experimental procedure was a slightly modified version of a procedure we used in two previous studies [41, 42]. The experimental material consisted of 54 bisyllabic words, all of which were used for the ongoing task; a subset of 10 (out of 54) words constituted the PM target stimuli. This 10-word subset did not differ from the overall 54 word set for frequency of occurrence in the Italian language [43]. Four experimental blocks were created; each block consisted of 48 trials of an ongoing task. Excluding the prospective target words (four prospective events in each block), there were 188 nontarget words in each block. A PC program randomly generated the four-word sequences of the ongoing trials with the following constraints: (i) the same word could not be repeated twice in a four-word sequence; (ii) the same sequence could not be repeated in each block and across blocks; (iii) the same word could not be repeated more than four times in each block. In two blocks, the ongoing task consisted of repeating forward a four-word sequence, and in the other two blocks it consisted in backward repeating of the sequences. In one block with forward repetition and one block with backward repetition of the sequences, the same target word was presented four times; in the remaining two blocks (one with forward and one with backward sequence repetition), four different target words were presented once. The target words were positioned pseudorandomly within the block by means of the following procedure: the 48 trials were divided into four parts, each consisting of 12 trials; the target word could appear randomly in each of the 12 trials and in any of the four positions of the word sequence. The percentage of sequences containing a target word was around 8% of the whole number of sequences in each block. In summary, across the four experimental blocks, two variables of interest were manipulated: the number of target words (in half of the blocks just one and in the other half four) and the instruction to repeat them in the ongoing verbal span task, which was forward in half of the blocks and backward in the other half.

**Figure 3:**
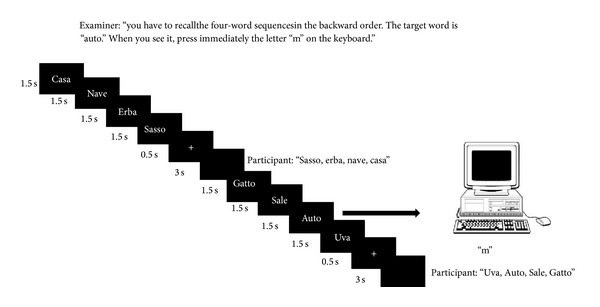
The figure reports two trials of the event-based PM task used in the present study. The first trial does not include a PM target whereas the second does. Each experimental block includes 48 trials. Four of them include a PM target (percentage of PM trials is around 8% of the whole trials). The example reported in the figure is from the experimental block with forward recall as the ongoing task and just one PM target word. In the other experimental blocks varied the order of recall in the ongoing task (forward versus backward) and/or the number of PM target words (1 versus 4).

At the beginning of each experimental block, the examiner informed the participants that they will be visually presented with sequences of four words that they have to repeat immediately after presentation in the forward or the backward modality, depending on the particular experimental block. Participants were also instructed that if during the sequence presentation one of the words was a target word they had to immediately press the “m” key on the computer keyboard. After that, the examiner read aloud the target word(s) for that particular block that the participant had to repeat immediately and after a delay of approximately one minute. To be sure that the participants had well understood and remembered the task instructions, after about two minutes of a resting phase they were required to repeat what they were expected to do in the experimental task and to recall the target word(s). The participants were also informed that the ongoing and the prospective tasks were equally important for obtaining a high score on the overall test. Then, the experimental PM procedure was run. Each of the four blocks of the experiment consisted of 48 consecutive trials. Each trial consisted of the visual presentation of four words (1.5 sec for each word with no interstimulus interval) in white letters in the centre of a black screen. A cross appearing at the centre of the screen for 0.5 sec signalled the end of the word sequence presentation. During a three sec. delay, during which the screen was blank and participants had to repeat the four-word sequence in the previously indicated order (forward or backward), the next sequence was presented. If one of the words in the sequence was a target word, they had to immediately press the “m” key on the computer keyboard (prospective task). At the end of each block, episodic memory for the target word(s) was assessed using a free recall procedure and a yes/no recognition procedure. The order of administration of the four blocks was randomized across subjects.  Figure 3 shows two sample trials and the overall organisation of the PM task.

The dependent variable for the ongoing task was the total number of correctly repeated four-word sequences. In the performance analysis of the PM task, we considered three different dependent variables: (i) the number of target words signalled, which represented an overall index of PM functioning, (ii) the number of target words recalled at the end of each block, which gave an index of the functioning of the retrospective component of PM, and (iii) the proportion of recalled or recognized words that elicited or did not elicit a prospective response that provided an index of PM contingent upon the declarative memory for the target words.

#### 2.4.2. Time-Based PM

The experimental material consisted of 4 groups of four actions each (e.g., “Switch on the light, turn off the PC, give a journal to the examiner and sign the current date on a paper”) that the patient was required to perform at the expiration of the established time. Other experimental material consisted of a series of paper and pencil exercises generally used in cognitive rehabilitation therapy (i.e., barrages of letters, numbers, or abstract symbols; searching for words; orthographic errors in a narrative), which were administered to the subjects as intercurrent tasks during the delay intervals of the PM task. The examiner and the experimental subject were seated at a table facing each other. The objects the subject had to use were on the table. A wall clock was placed to the right of the subject so that he had to turn his head to check it.

At the beginning of each session, the examiner instructed the participant to perform four different actions after 15 min. had elapsed. If the patient claimed he did not understand what he was supposed to do, the examiner repeated the instructions to be sure the patient understood them. Immediately afterwards, the participant performed the intercurrent tasks. When the 15 min. were up, the examiner noted the actions carried out spontaneously by the individual. There was a 2 min. tolerance limit before and after time expiration during which the individual could initiate the prospective task. If the patient still did not show that he remembered having to carry out some action 2 min. after the time had expired, he was reminded by the examiner (“Do you remember that at this point you were supposed to do something?”). In the case of an affirmative response, the examiner recorded the number of actions carried out correctly. Thus, for each trial of the experimental task two distinct scores were given, one for recall of the intention to perform the actions (prospective component) (maximum score: 4) and another for correct execution of the actions (retrospective component) (maximum score: 16). In the former case, a score of 1 was given for each intention activated and a score of 0 if the intention was absent or the activation was incorrect (i.e., the subject activated the intention in a wrong temporal window). In the latter case, a score of 1 was attributed to any action correctly performed, a score of 0.5 to a partially correct action, and a score of 0 to a lacking or completely incorrect action.

## 3. Results

G.P.'s performances were compared with those of 8 healthy individuals matched for age, years of formal education, and gender. Similarly, V.B.'s performances were compared with those of 7 healthy men matched for age and education. For the statistical analysis, we used Crawford and Garthwaite's procedure [44] which allows evaluating whether an individual's score is significantly different from a control or normative sample.

### 3.1. Neuropsychological Evaluation

Results of the two patients and relative control groups on the general neuropsychological battery are summarized in Table 1.

Both patients obtained normal scores on the Coloured Progressive Matrices, the span tests, and the visuospatial tests. Patient V.B. also performed normally on the declarative memory tests. Patient G.P., instead, performed poorly on the verbal episodic memory tests. Indeed, he performed below the range of normal controls in the immediate and delayed recall of both the word list and the prose and in recognizing the word list.

The scores of the two patients and the normal controls on the executive battery tests are reported in Table 2.

V.B. performed normally on all tests, whereas G.P. performed abnormally slowly on several tests of the executive battery. Indeed, he required more time than normal controls on the A and B subtests of the Trail Making Test (but the critical difference between the two subtests was in the normal range), the word reading, colour naming, and critical condition of interference on the Stroop test and in several planning and execution conditions of the Zoo Map test. Moreover, he performed below normal on the phonological, semantic, and alternate versions of the Word Fluency task.

### 3.2. Prospective Memory Assessment

#### 3.2.1. Event-Based PM

Results of patients and normal controls on the event-based prospective memory task are summarized in Table 3.

Overall, the two patients were less accurate than NCs in signalling the appearance of the target words. G.P.'s scores were abnormal on both the forward tasks (one- and four-word sequences) and the one-word sequence of the backward task. V.B., instead, performed below normal only on the one-word sequence of the backward test. However, when accuracy was expressed as the sum in all test conditions, both patients performed below the normal controls' range.

The patients' poor accuracy on the prospective memory task could not be ascribed to failure to remember the target words. Both patients performed in the normal range on the tests of free recall and recognition of target words. This was particularly true for V.B., whose scores were in the higher normal range in all conditions of the experimental task. G.P., instead, recalled or recognized the four target words in the lower range of the controls, and the sum of his remembered words across the various test conditions was marginally different from controls.

To further explore the hypothesis that reduced accuracy on the prospective memory task was not an epiphenomenon of poor declarative memory for the target words (at least in the case of G.P.), we calculated whether the proportion of recalled or recognized words that elicited or did not elicit a prospective response (during the PM task) was the same or different in the two patients and NCs. A similar proportion of prospective hits among words that were subsequently recalled or recognized would suggest that the retrospective memory deficit (i.e., a declarative memory loss for the target words) underlies the reduced accuracy on the PM task. Conversely, a lower proportion of prospective hits among the recalled or recognized words in the patients than in the NC group would suggest that the PM deficit was due to reduced ability to activate the prospective intention even for words whose declarative memory was substantially preserved. Indeed, in the four-word test 16.7% of the correctly recalled or recognized words elicited a PM response from V.B., whereas this proportion was around 70% for the NC group. For G.P. this proportion was 0% for both recalled and recognized words and was around 80% in the NC group. In the one-word test, 62% of the recalled or recognized words elicited a PM response from V.B and 93% elicited a PM response from the NC group. Moreover, 50% of the recalled words elicited the PM response in G.B. and 86% in the NC group. Conversely, the number of target words that were not recalled or recognized but, nevertheless, elicited a PM response was overall low and substantially the same in the patients (around 13% and 0% in the four-word and one-word tasks, resp.) and NCs (around 15% and 5% in the four-word and one-word tasks, resp.). These results clearly indicate that a reduced declarative memory for target words could not account for the PM deficit exhibited by the two patients. Conversely, difficulty in reactivating the planned intention at the appearance of the target event was at the core of their prospective memory deficit.

#### 3.2.2. Time-Based PM

The two patients were clearly impaired on the prospective component of the memory task. Indeed, G.P. did not spontaneously initiate any action when the time expired (NCs: 3.62 ± 0.74; *t* = 4.62; *P* < .001) and V.B. remembered that some actions had to be carried out only in one case (NCs: 3.62 ± 1.06; *t* = 2.33; *P* = .026). Instead, the two patients exhibited no deficit on the retrospective component of the task. In fact, after the examiner's solicitation, G.P. correctly executed 15 actions (NCs: 15.3 ± 0.8; *t* = 0.34; *P* = .37) and V.B. correctly recalled 13.5 actions either spontaneously or after solicitation (NCs: 14.4 ± 1.5; *t* = 0.54; *P* = .30).

## 4. Discussion

Here we report two patients with focal damage in the prefrontal regions of the brain resulting from severe accidental brain injury. Both patients reported that a severe deficit in fulfilling delayed intentions during the activities of daily living was their most recognizable and disabling cognitive deficit. The PM impairment was confirmed in a laboratory setting, where the two patients performed abnormally on both an event-based and a time-based experimental procedure. In both patients, results of these two tasks revealed dissociation between a deficit in the prospective component and sparing of the retrospective component of PM. Indeed, they failed to activate the prospective intention following the occurrence of the target event or when the established time had elapsed. When questioned by the examiner, however, they correctly recalled either the target words (in the event-based task) or the specific actions to be performed (in the time-based task). Moreover, an analysis of the correspondence between the target words that were successively remembered and those that were able to elicit a PM response during the event-based PM task clearly indicated that the patients had difficulty in spontaneously activating the prospective intention even though they had normal declarative memory of the target words.

Despite these apparent similarities, several aspects of the qualitative profile of cognitive impairment distinguish the two patients. In V.B. the PM deficit occurred in the context of substantially preserved cognitive functions. As the analysis of executive and declarative memory abilities was particularly detailed, we were quite confident that the patient had no problems with planning, set-shifting, resistance to interference and working memory, or long-term memory for structured and unstructured verbal material and visuospatial data. By contrast, G.P.'s cognitive deficit involved the executive functions pervasively, with the only possible exception of working memory. In fact, G.P. was impaired in plan formation (Zoo Map test), verbal fluency (both phonological and categorical), and selective attention (Stroop test). As for set-shifting, he performed in the normal range on the WCST (i.e., for both categories achieved and number and quality of errors) and demonstrated normal slowing when performing version B with respect to version A of the TMT, but he was severely impaired on the alternate Verbal Fluency task. G.P.'s declarative memory deficit is particularly relevant in light of the previously described sparing of the retrospective component of PM. Indeed, as previously noted, it is generally acknowledged that a declarative memory deficit underlies impairment of the retrospective component of a PM task [6, 14]. Working memory processes could have contributed to the maintenance of target words in the event-based task and, as noted, working memory was likely G.P.'s best preserved executive function. The delay interval in the time-based task was long enough for the retention of planned actions to require declarative memory processes. The memory load, however, was less demanding (four items had to be retained), so G.P.'s reduced memory resources might have been sufficient to manage them.

In a neurocognitive perspective, the most interesting result of the present study is the evidence that in a patient with frontal damage, the PM deficit could be observed either in the context of a pervasive deficit of executive and declarative memory functions or as an isolated cognitive disorder. In G.P., the simultaneous presence of a PM impairment and of deficient performance on tests assessing a variety of executive domains might suggest a putative causal relationship. Indeed, the patient's planning deficit could have interfered with his ability to formulate a plan of future actions. This may have been less important in the laboratory tests of PM, which offer the plan of action with the test instructions but could have been critical for the PM failures in daily living, in which articulate plans of action are frequently required to meet temporal and environmental constraints [45]. The selective attention deficit could have made the simultaneous management of the ongoing task and the prospective procedure (to rehearse the intention and to monitor the passing of time) problematic, thus resulting in accelerated forgetting of the intention and missing the critical temporal window for the time-based tasks [46]. Finally, the set-shifting deficit could have made the mental approach more rigid towards complex situations in which performance of intercurrent tasks had to be conciliated with the need to respond to target events or elapsing time to fulfill the delayed intentions [17].

The logic of inferring causal relationships between concomitant cognitive deficits has been criticized because the coexistence of deficits does not guarantee a functional relationship. In fact, the case of V.B. shows that a PM impairment can be observed in a patient whose executive functions (in the various subdomains of planning, selective attention, set-shifting, and working memory) are largely preserved, as demonstrated by his high level performances in a variety of ad hoc tests. However, the case of V.B. is unlike previously reported cases in which a PM impairment was apparent only in very demanding tasks, consistent with a deficit in planning and managing multitask situations [19, 20]. In fact, V.B. scored in the normal range on a test (the Zoo Map) that required simultaneously considering a multiplicity of spatial and temporal constraints to achieve an adequate plan of action. More importantly, however, this patient failed on PM tasks (both time-based and event-based) that did not require formulating a plan of action but were largely heterodirected and simply involved obeying instructions. In other words, V.B.'s PM deficit is not the expression of difficulty in planning or managing complex multitask situations but is more basically related to an impairment in activating the prospective intention to act when the context or the time meet the encoded plan.

To answer to the question posed in the paper title, that is, if there is a causal relationship between prospective memory impairment and executive dysfunction in prefrontal lobe damaged patients, the result of the present investigation (together with other neuropsychological evidence of the literature) suggests a quite problematic response. Indeed, the bulk of neuropsychological evidence is that a significant correlation exists between PM performance and some kind of executive deficits [16–21] and the case of G.P. adds to this evidence reporting concomitant PM and executive impairment. This relationship could be actually a simple epiphenomenon, due to the topographical contiguity of cerebral areas involved in the executive and PM functions, rather than the expression of a true functional relationship. Alternatively, in view of the complex nature of a PM task, in which planning, working memory, and attentional and set-shifting abilities evidently play a significant role, such a correlation could be genuine, disclosing a real functional dependence of PM deficits over an executive impairment. On the other side, the existence of patients with a “pure” PM deficit (such as V.B.), in which a clear dissociation exists between PM impairment and preserved executive functions, demonstrates that such a functional relationship is not obligatory, but that a PM impairment could be the result of more basic cognitive deficits, which specifically regard the abilities which are critical for the fulfilment of delayed intentions.

In a neurobiological perspective, V.B.'s BA10 damage suggests that the PM deficit should be interpreted in terms of failure to coordinate responses to stimulus-independent and stimulus-oriented tasks. Burgess et al. [47] proposed that the interplay between these fundamental aspects of attention is critical when subjects intend to carry out previously formulated intentions. Stimulus-oriented attending enhances our ability to notice changes in the environment when attention is oriented toward external stimuli. Instead, stimulus independent attending occurs when attention is directed toward self-generated thoughts. In fact, during a PM task attention is continuously biased between stimulus-oriented attending for the target cue or elapsing time detection and stimulus-independent attending for access to the characteristics of the intended actions. It has been proposed that BA 10 contributes to the normal functioning of this interplay by acting as a sort of “gateway” [47]. Functional neuroimaging evidence supports the view that the lateral portion of BA 10 is mainly involved in stimulus independent attending and that medial BA 10 mediates stimulus-oriented attending [48, 49]. The lesion of BA 10 (both lateral and mesial portions) could have interfered with this mechanism, leaving the patient unable to appropriately alternate his focus of attention toward stimulus-oriented and stimulus-independent attending.

As for localization of the cerebral damage, both patients presented a rather large contusive lesion involving BA 10 and various adjacent cortical regions. The main difference between the patients was the hemispheric side of the prevalent damage: V.B.'s contusive lesion was confined to the most rostral regions of the right hemisphere and G.P.'s damage was bilateral but prevalently on the left side. Data reported in the literature are not univocal regarding a possible differential role of the right and left frontal lobes in PM functioning. Recent reviews of functional neuroimaging data failed to find a consistent pattern of hemispheric lateralization in both time-based and event-based PM experiments [28, 47]. In fact, recent neuropsychological data seem to suggest that right-sided frontal lesions are more consistently associated with PM failure. In a study involving 74 focal brain-damaged individuals, a discriminant function analysis with 12 areas of possible cortical damage as independent variable revealed that lesions in the right dorsolateral prefrontal cortex mostly contributed to predicting performance on a PM task [26]. Similarly, a study based on a voxel-based analysis of focal brain lesions in 45 individuals found that damage in the right polar prefrontal region (in Brodmann area 10) was specifically associated with a deficit in time-based prospective memory tasks for both words and pictures [27]. Therefore, the selective PM deficit we found in patient V.B. could be related to the special role of polar and dorsal frontal regions of the right hemisphere in PM. It should be noted, however, that in two recent studies of healthy subjects in which the role of the frontal pole in PM functioning was investigated using transcranial magnetic stimulation, the authors found differential left versus right involvement as a function of the material employed in the PM procedure (i.e., words versus spatial location, resp.) [41, 50].

As a final clinical comment, it should be noted that different localization and extension of brain damage in the two cases may only partially explain the qualitative and quantitative differences in the neuropsychological profiles exhibited by the two patients. Indeed, the time elapsed from head trauma and the cognitive assessment reported here are significantly different in the two patients. G.P. suffered brain injury approximately one year before our neuropsychological investigation, and V.B.'s traumatic brain injury occurred 17 years before the neuropsychological exam. We can hypothesize that to recover from his cognitive impairments V.B. benefited more than G.P. from brain plasticity processes. In this context, the finding that V.B. presented with a selective deterioration of PM functioning is particularly relevant, because it suggests that this ability is specifically susceptible to brain damage. Indeed, V.B. might not have suffered from a significant executive dysfunction and in this case the PM impairment might be his only significant deficit or, alternatively, he might have recovered from an initial executive deficit and, in this case, the PM failure might have been his only residual cognitive deficit. The high sensitivity of PM tasks to cognitive decline is confirmed in the literature on patients in the prodromal phase of dementia syndromes. Indeed, data have been reported which demonstrate that measures of PM functioning discriminate better between individuals with a diagnosis of Mild Cognitive Impairment and healthy controls than traditional tests of declarative memory and executive functions [42]. These observations should directly inform clinical practice by indicating that the administration of PM procedures might better capture the cognitive consequence of brain injury.

## Figures and Tables

**Table 1:** Performance scores of G.P. and V.B. on the tests of neuropsychological battery. *t*-tests for significant differences of individual scores from average scores of groups of normal controls are also reported.

	G.P.	Healthy controls (*N* = 8) M (SD)	*t*	*P*	V.B.	Healthy controls (*N* = 8) M (SD)	*t*	*P*
General intelligence								
Raven's Coloured Matrices(Carlesimo et al., 1996, [31])	35	35.0 (0.9)	0.00	.50	34	34.1 (2.5)	0.03	.48
Short-term memory								
Digit span forward (Orsini et al., 1987, [33])	6	7.3 (1.2)	1.00	.17	7	6.7 (1.0)	0.23	.41
Corsi span forward (Orsini et al., 1987, [33])	7	6.4 (0.5)	1.1	.15	6	6.4 (0.7)	.48	.32
Declarative memory								
15-word learning task (Carlesimo et al., 1996, [31])								
Immediate recall	39	54.6 (7.8)	1.89	.05*	44	51.1 (6.1)	1.10	.15
15 min. delayed recall	5	12.5 (1.9)	3.72	.004*	10	13.1 (1.8)	1.62	.07
Recognition (hit rates + correct rejections)	24	29.6 (0.5)	10.2	.001*	28	29.0 (1.4)	0.67	.26
Prose recall (Carlesimo et al., 2002, [32])								
Immediate recall	4.1	6.9 (0.8)	3.43	.006*	6.6	6.5 (1.0)	0.09	.46
20 min. delayed recall	4.1	6.9 (0.7)	3.62	.004*	6.3	6.7 (1.3)	0.29	.39
Rey's Figure (Carlesimo et al., 2002, [32])								
Immediate reproduction	21	26.2 (4.0)	1.26	.12	18	25.8 (7.3)	1.01	.17
15 min. delayed reproduction	22	27.3 (3.5)	1.43	.10	18	25.5 (6.1)	1.16	.14
Supraspan spatial sequence learning (Spinnler and Tognoni, 1987, [34])	26.7	27.2 (1.3)	0.36	.36	26.6	26.7 (2.9)	0.03	.49
Visuospatial abilities								
Copy of drawings (Carlesimo et al., 1996, [31])	10	10.7 (1.0)	0.66	.27	12	11.5 (0.9)	0.52	.31
Copy of drawings with Landmarks (Carlesimo et al., 1996, [31])	69	69.0 (2.8)	0.00	.50	70	70 (0.0)	0.00	.50
Rey's Figure Copy (Carlesimo et al., 2002, [32])	30	35.0 (2.8)	1.68	.07	36	35.2 (1.8)	0.42	.34

*Significant difference.

**Table 2:** Performance scores of G.P. and V.B. on the tests of executive battery. *t*-tests for significant differences of individual scores from average scores of groups of normal controls are also reported.

	G.P.	Healthy controls (*N* = 8) M (SD)	*t*	*P*	V.B.	Healthy controls (*N* = 8) M (SD)	*t*	*P*
Modified card sorting test (Nocentini et al., 2002, [35])								
Criteria achieved	6	5.9 (0.4)	0.24	.41	6	5.9 (0.4)	0.24	.41
Perseverative Errors	0	1.0 (2.4)	0.39	.35	0	0.9 (1.6)	0.53	.30
Nonperseverative errors	6	3.3 (2.3)	1.10	.15	0	1.5 (1.4)	1.01	.17
Trail making test (Giovagnoli et al., 1996, [36])								
A (sec.)	53	26.2 (12.4)	2.03	.04*	40	29.0 (12.2)	0.85	.21
B (sec.)	110	72.5 (17.9)	1.97	.04*	60	73.3 (19.6)	0.64	.27
B − A (sec.)	57	46.3 (16.5)	0.62	.28	20	44.3 (15.9)	1.44	.10
Stroop test (Stroop, 1935, [38])								
Word reading								
Accuracy	50	73.5 (14.4)	1.54	.08	77	72.6 (15.9)	0.26	.40
Time (sec.)	65	44.1 (8.9)	2.22	.03*	39	42.4 (10.7)	0.30	.39
Colour naming:								
Accuracy	50	52.9 (5.0)	0.55	.30	71	54.3 (8.6)	1.83	.06
Time (sec.)	74	61.1 (5.0)	2.43	.02*	55	63.0 (20.0)	0.38	.36
Colour naming with interference								
Accuracy	23	30.0 (6.5)	1.01	.17	45	33.0 (9.1)	1.24	.13
Time (sec.)	110	95.0 (6.6)	2.14	.03*	96	105.8 (41.5)	0.22	.41
Zoo Map test (Wilson et al., 1998, [39])								
Version 1								
Planning time (sec.)	260	126.3 (53.1)	2.37	.02*	228	136.1 (102.9)	0.84	.21
Execution accuracy	8	5.4 (3.5)	0.70	.25	3	6.5 (2.4)	1.37	.11
Execution time (sec.)	72	49.6 (19.0)	1.51	.09	21	41.3 (26.9)	0.71	.25
Version 2								
Planning time (sec.)	37	20.6 (14.1)	1.10	.15	45	29.0 (14.7)	1.03	.17
Execution accuracy	8	8.0 (0.0)	0.0	.50	7	7.1 (2.1)	0.04	.48
Execution time (sec.)	52	21.9 (4.6)	6.12	.001*	30	27.4 (18.1)	0.14	.45
Phonological verbal fluency (Carlesimo et al., 1996, [31])	15	36.0 (9.0)	2.22	.03*	49	41.4 (9.6)	0.74	.24
Semantic verbal fluency (Spinnler and Tognoni, 1987, [34])	43	62.4 (10.6)	1.73	.06	67	63.1 (9.4)	0.39	.35
Alternate verbal fluency (Henry and Crawford, 2004, [37])	6	21.5 (4.9)	2.98	.01*	24	20.3 (3.9)	0.89	.20
Digit span backwards (Monaco et al., 2013, [40])	6	5.8 (0.7)	0.34	.37	4	5.9 (1.0)	1.80	.06
Corsi span backwards (Monaco et al., 2013, [40])	6	5.8 (0.9)	0.27	.40	6	6.3 (1.3)	.18	.43

*Significant difference.

**Table 3:** Performance scores of G.P. and V.B. on the event-based PM task. *t*-tests for significant differences of individual scores from average scores of groups of normal controls are also reported.

	G.P.	NCs (*N* = 8) M (SD)	*t*	*P*	V.B.	NCs (*N* = 8) M (SD)	*t*	*P*
Number of target words signaled during each block								
Forward ongoing task								
1 target word	2	3.5 (0.8)	1.89	.05*	3	3.8 (0.5)	1.54	.08
4 target words	1	3.5 (0.8)	3.14	.01*	1	2.3 (1.8)	0.67	.26
Backwards ongoing task								
1 target word	2	3.4 (0.5)	2.53	.02*	2	3.8 (0.5)	3.59	.01*
4 target words	1	1.8 (1.5)	0.48	.32	0	1.9 (1.2)	1.43	.10
Total	**6**	**12.1 (2.2)**	**2.59**	.02*	**6**	**11.6 (2.3)**	**2.28**	.03*

Number of target words recalled at the end of each block								
Forward ongoing task								
** ** 1 target word	1	1.0 (0.0)	0.00	.50	1	1.0 (0.0)	0.00	.50
** ** 4 target words	2	2.9 (1.1)	0.73	.24	4	2.6 (1.7)	0.77	.23
Backwards ongoing task								
1 target word	1	1.0 (0.0)	0.00	.50	1	1.0 (0.0)	0.00	.50
** ** 4 target words	0	2.2 (1.5)	1.43	.10	2	2.0 (1.1)	0.00	.50
Total	**4**	**7.1 (2.0)**	**1.45**	**.10**	**8**	**6.6 (2.3)**	**0.58**	**.29**

Number of target words recognized at the end of each block								
Forward ongoing task								
** ** 1 target word	1	1.0 (0.0)	0.00	.50	1	1.0 (0.0)	0.00	.50
** ** 4 target words	2	3.3 (1.0)	1.18	.14	3	2.6 (1.7)	0.22	.41
Backwards ongoing task								
** ** 1 target word	1	1.0 (0.0)	0.00	.50	1	1.0 (0.0)	0.00	.50
** ** 4 target words	1	2.8 (1.4)	1.19	.14	3	2.4 (1.1)	0.51	.31
Total	**5**	**8.0 (1.9)**	**1.49**	**.09**	**8**	**7.4 (3.1)**	**0.18**	**.43**

*Significant difference.
